# The predictive value of current haemoglobin levels for incident tuberculosis and/or mortality during long-term antiretroviral therapy in South Africa: a cohort study

**DOI:** 10.1186/s12916-015-0320-9

**Published:** 2015-04-02

**Authors:** Andrew D Kerkhoff, Robin Wood, Frank G Cobelens, Ankur Gupta-Wright, Linda-Gail Bekker, Stephen D Lawn

**Affiliations:** George Washington University School of Medicine and Health Sciences, 2300 Eye St, NW, Washington, DC 20037 USA; Department of Global Health, Academic Medical Center, Amsterdam Institute for Global Health and Development, University of Amsterdam, Amsterdam, the Netherlands; The Desmond Tutu HIV Centre, Institute of Infectious Disease and Molecular Medicine, Faculty of Health Sciences, University of Cape Town, Cape Town, South Africa; Department of Clinical Research, Faculty of Infectious and Tropical Diseases, London School of Hygiene and Tropical Medicine, London, UK; KNCV Tuberculosis Foundation, The Hague, the Netherlands

**Keywords:** HIV, AIDS, Tuberculosis, Africa, Anaemia, Antiretroviral, Mortality

## Abstract

**Background:**

Low haemoglobin concentrations may be predictive of incident tuberculosis (TB) and death in HIV-infected patients receiving antiretroviral therapy (ART), but data are limited and inconsistent. We examined these relationships retrospectively in a long-term South African ART cohort with multiple time-updated haemoglobin measurements.

**Methods:**

Prospectively collected clinical data on patients receiving ART for up to 8 years in a community-based cohort were analysed. Time-updated haemoglobin concentrations, CD4 counts and HIV viral loads were recorded, and TB diagnoses and deaths from all causes were ascertained. Anaemia severity was classified using World Health Organization criteria. TB incidence and mortality rates were calculated and Poisson regression models were used to identify independent predictors of incident TB and mortality, respectively.

**Results:**

During a median follow-up of 5.0 years (IQR, 2.5-5.8) of 1,521 patients, 476 cases of incident TB and 192 deaths occurred during 6,459 person-years (PYs) of follow-up. TB incidence rates were strongly associated with time-updated anaemia severity; those without anaemia had a rate of 4.4 (95%CI, 3.8-5.1) cases/100 PYs compared to 10.0 (95%CI, 8.3-12.1), 26.6 (95%CI, 22.5-31.7) and 87.8 (95%CI, 57.0-138.2) cases/100 PYs in those with mild, moderate and severe anaemia, respectively. Similarly, mortality rates in those with no anaemia or mild, moderate and severe time-updated anaemia were 1.1 (95%CI, 0.8-1.5), 3.5 (95%CI, 2.7-4.8), 11.8 (95%CI, 9.5-14.8) and 28.2 (95%CI, 16.5-51.5) cases/100 PYs, respectively. Moderate and severe anaemia (time-updated) during ART were the strongest independent predictors for incident TB (adjusted IRR = 3.8 [95%CI, 3.0-4.8] and 8.2 [95%CI, 5.3-12.7], respectively) and for mortality (adjusted IRR = 6.0 [95%CI, 3.9-9.2] and adjusted IRR = 8.0 [95%CI, 3.9-16.4], respectively).

**Conclusions:**

Increasing severity of anaemia was associated with exceptionally high rates of both incident TB and mortality during long-term ART. Patients receiving ART who have moderate or severe anaemia should be prioritized for TB screening using microbiological assays and may require adjunctive clinical interventions.

## Background

Although antiretroviral therapy (ART) reduces tuberculosis (TB) incidence rates by approximately 70% in people living with HIV [1], high TB incidence rates continue to be observed among patients receiving ART in sub-Saharan Africa, especially during the initial months of treatment [2-9]. Moreover, in patients receiving ART long-term, TB incidence rates remain substantially higher than background rates, even in those with good immune recovery [2]. Incident TB also remains an important independent risk factor for mortality during short-term and long-term ART [4,10]. To improve clinical outcomes in ART programmes in sub-Saharan Africa, it is important to identify risk factors and/or predictive markers associated with incident TB and mortality so that effective interventions may be designed and implemented.

Measurement of haemoglobin concentrations is low-cost, more widely available than CD4 cell count measurement in clinical settings in sub-Saharan Africa, and may have predictive value for both active TB disease and all-cause mortality. We have previously demonstrated that greater degrees of anaemia severity at ART initiation are associated with a high prevalence of undiagnosed TB, as well as death during the initial months of ART [11]. While it remains poorly defined, we hypothesized that time-updated haemoglobin concentrations may be associated with incident TB and/or death during long-term ART. Therefore we undertook this retrospective cohort analysis among South African patients receiving ART for up to 8 years to determine TB incidence and mortality rates stratified by time-updated anaemia severity. We also sought to determine whether time-updated anaemia severity was an independent predictor for incident TB and/or mortality even after adjustment for time-updated CD4 counts.

## Methods

### Study setting and data collection

A retrospective cohort analysis was undertaken among patients recruited for previously reported studies [2,10] and was conducted as part of ongoing research at the Hannan Crusaid Antiretroviral Centre in Gugulethu township, Cape Town, South Africa [6,12,13]. All consecutively recruited ART-naïve adult patients (age ≥16 years) who enrolled in the ART programme and subsequently started ART were eligible for study inclusion. All patients provided written informed consent. This study was approved by the human ethics committee of the University of Cape Town, Cape Town, South Africa and was reported in conformity with the STROBE Statement checklist for cohort studies [14].

A total of 2,000 patients enrolled between September 2002 and May 2006 (an interval for which very complete clinical records and time-updated TB, CD4 count and viral load data were available) and were followed up until censoring of observations on 01 January 2011. During this period, patients were eligible to start ART if they had either a CD4 count <200 cells/uL or a World Health Organization (WHO) stage 4 disease, which was in accordance with national ART guidelines at that time. First-line ART comprised a non-nucleoside reverse transcriptase inhibitor (predominantly efavirenz) with a nucleoside reverse transcriptase inhibitor backbone (stavudine or zidovudine and lamivudine), while the second-line regimen was protease inhibitor-based (lopinavir/ritonavir) with a nucleoside reverse transcriptase inhibitor backbone (zidovudine and didanosine). Information on the ART regimen was attained from electronic pharmacy records. All patients received daily trimethoprim-sulphamethoxazole prophylaxis. However, in accordance with local guidelines and practice at the time, patients did not receive isoniazid preventive therapy (IPT). All treatment was provided free of charge.

Primary care clinics, TB clinics and antenatal clinics referred patients for ART, and upon a patient’s first visit to the clinic their demographic details were recorded. All patients received routine clinical review at a screening visit, ART initiation, after 4, 8 and 16 weeks of ART, and at least every 4 months thereafter. Haemoglobin concentrations as well as blood CD4 cell counts and plasma HIV viral load levels were measured routinely before starting ART, every 4 months during ART and as clinically indicated. Laboratory results were transferred on a monthly basis to an electronic database. All information related to TB diagnoses and treatment was obtained from prospectively maintained structured clinical records.

### TB screening and diagnosis

All patients were screened for TB prior to starting ART using a multi-symptom screening questionnaire to identify patients who required further TB investigations. Several investigations were available to diagnose TB either pre-ART or during ART, including sputum induction, sputum smear fluorescence microscopy, automated liquid culture of sputum using mycobacterial growth indicator tubes (MGIT 960, Becton Dickinson, Sparks, Maryland, USA), chest radiology, ultrasonography and fine needle lymph node aspiration cytology. TB case ascertainment approaches were similar for the duration of the study period, and all follow-up was completed prior to the national scale-up of Xpert MTB/RIF assay in South Africa. All TB cases fulfilled the WHO diagnostic criteria for resource-constrained settings with high HIV prevalence. While the large majority of cases were culture-proven, any culture-negative TB diagnoses were defined by strong clinical or histological evidence of active TB, radiological abnormalities consistent with active TB and the clinician’s decision to treat with a full course of anti-TB therapy. All microbiological specimens were analysed in accredited laboratories using standardized methods, and all positive cultures of mycobacteria were speciated by polymerase chain reaction. TB treatment was rifampicin-based and administered via a local network of community clinics using the directly observed treatment short-course (DOTS) strategy.

### Definitions

Baseline and time-updated haemoglobin levels were used to classify anaemia severity according to WHO criteria [15]: no anaemia (haemoglobin concentration ≥13.0 g/dL for males, ≥12.0 g/dL for females), mild anaemia (11.0-12.9 g/dL for males, 11.0-11.9 g/dL for females), moderate anaemia (8.0-10.9 g/dL for males and females) or severe anaemia (<8.0 g/dL for males and females). ‘Baseline haemoglobin level’ was defined using a haemoglobin concentration within 90 days of starting ART for those without a known TB diagnosis prior to ART initiation (and currently receiving anti-TB therapy) and within 30 days for those with known TB who were currently receiving anti-TB therapy, as it is known to be associated with rapid changes in haemoglobin concentration [16-18].

A ‘past history of TB’ was defined by those who had previously been diagnosed with TB and had completed a full course of anti-TB therapy prior to study enrolment. ‘Prevalent TB’ was defined by any patient with a known TB diagnosis who was currently receiving anti-TB therapy at the time of ART initiation. Any new clinical episode of TB diagnosed after starting ART, irrespective of date of symptom onset, was defined as ‘incident TB.’ ‘Recurrent TB’ diagnoses were made with reference to any previous episodes of TB, including those made prior to ART enrolment (that is, past history of TB, prevalent TB or incident TB). Patients who had multiple incident TB diagnoses completed full courses of anti-TB therapy and had complete resolution of symptoms between episodes. Thus, patients undergoing retreatment after failure of initial treatment or those returning after treatment default did not count towards a case of incident TB or recurrent TB.

### Clinical outcomes

We determined clinical outcomes of patients during routine follow-up within the ART service as previously described [19]. Patients’ outcomes were classified into one of four mutually exclusive groups: alive within the programme and receiving ART, transfer to another ART service, death from any cause, or lost to follow-up (LTFU). LTFU was defined by those receiving ART who were more than 12 weeks late for a scheduled clinic appointment or who failed to return to the ART programme, and could not be located by active community-based follow-up.

### Data analysis

Data were analysed using Stata version 12.0 (College Station, TX). Analysis was restricted to those who had at least one haemoglobin result available from any point either prior to ART initiation or during ART. If either a haemoglobin level or a CD4 cell count was missing for 1 or more intervals, a mean of the values for the preceding and subsequent intervals was used (about 1.5% intervals). Chi-squared tests and Kruskall-Wallis tests were used to compare proportions and medians, respectively. Since we analysed existing data, we did not undertake formal sample size calculations. However, the net sample size of n = 1,521 provided a statistical power of close to 1.0 for most expected outcomes, and 0.88 for the extreme case of finding an only twofold difference (10 versus 5/100 person-years, PY) in TB incidence between equal groups with and without anaemia. All statistical tests were two-sided at α = 0.05.

Person-time was divided into intervals predefined by haemoglobin results at the start of each interval that were done at least 4-monthly and started accruing from the date of ART initiation and continued until death, transfer-out, LTFU, or data censorship on 01 January 2011. Haemoglobin levels and CD4 counts were coded as time-dependent covariates, and these values were updated at least every 4 months as a repeated measure. Only intervals with both a time-updated haemoglobin level and CD4 result counted towards person-time accrued. TB incidence and mortality rates with corresponding 95% confidence intervals stratified according to time-updated WHO anaemia severity and CD4 cell strata were calculated. This meant that a patient could be reclassified at each time-updated interval on the basis of their current anaemia severity or CD4 count and that a case of incident TB or death was only counted for that person-time observation if it occurred during that period. Person-time accrued during treatment of prevalent or incident TB was excluded when calculating TB incidence rates. Multivariable models to determine factors associated with incident TB and mortality during ART were constructed using Poisson regression. A gamma distributed random-effects model was used to adjust for repeated measurements in patients with incident TB. A priori covariates (time-updated anaemia severity and time-updated CD4 cell count) and variables meeting the predefined cutoff of *P* ≤ 0.1 in the univariable model were included in the multivariable model. Likelihood ratio tests were used to investigate interactions between variables and test statistical hypotheses. Sensitivity analyses were undertaken to determine if the relationship between time-updated anaemia severity and mortality rates (unadjusted and adjusted) was affected by patients LTFU where it was assumed that all patients LTFU had died.

## Results

**Figure 1:**
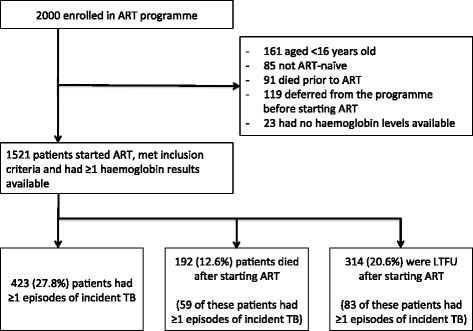
**Study overview including diagnoses of incident TB and patient outcomes.**

From September 2002 to May 2006, 2,000 patients were enrolled into the ART service and 1,544 met inclusion criteria, although 23 patients were excluded due to lack of any haemoglobin measurements (Figure 1). Thus, 1,521 patients were included in the final analysis and accrued 6,459 person-years of follow-up in the ART service for a median of 5.0 years (IQR, 2.5-5.8; range 0.0-8.2). During ART, 192 (12.6%) patients died, 314 (20.6%) were LTFU, 205 (13.5%) transferred to a different ART service and 810 (53.3%) were alive at the time that data were censored.

At baseline, patients had a median age of 33 years and more than two-thirds were female (Table 1). Advanced immunodeficiency was common; the median CD4 count was 101 cells/uL and more than 80% had stage 3 or 4 disease. The median haemoglobin was 11.2 g/dL (IQR, 9.8-12.5) and 72% had anaemia of which 44% had moderate or severe anaemia. Nearly half of all patients had a past medical history of TB upon enrolment and 30% were receiving anti-TB therapy at the time of ART initiation (Table 1). The baseline characteristics (including haemoglobin level and CD4 cell count) of those who were LTFU (n = 314) or transferred their care to another ART service (n = 205) did not differ from those who remained alive and in programme (n = 810), with the exception that patients LTFU tended to be slightly younger and have prevalent TB (Table 1).

**Table 1:** **Baseline characteristics of patients stratified according to outcome (n = 1,521)**

	**All (n = 1,521)**	**Alive (n = 810)**	**Died (n = 192)**	**LTFU (n = 314)**	**Transfer-out (n = 205)**	***P*** **-value** ^**¥**^
**Age (at baseline), median (IQR)**	33 (28-33)	33 (29-38)	35 (29-43)	31 (27-36)	32 (28-38)	<0.001^#^
**Gender**						
Male	451 (29.7)	235 (29.0)	77 (40.1)	92 (29.3)	47 (22.9)	0.002
Female	1,070 (70.4)	575 (71.0)	115 (59.9)	222 (70.7)	158 (77.1)	
**WHO disease stage (at baseline)**						
1 or 2	302 (19.9)	184 (22.7)	14 (7.3)	64 (20.4)	40 (19.5)	<0.001
3 or 4	1219 (80.1)	626 (77.3)	178 (92.7)	250 (79.6)	165 (80.5)	
**CD4 cell count (cells/μL), median, IQR** ^**a**^	101 (49-158)	102 (53-160)	77 (25-138)	108 (53-168)	107 (48-151)	<0.001
**CD4 category**						
≥200	173 (11.6)	98 (12.4)	15 (7.9)	39 (12.8)	21 (10.5)	0.072
100-199	582 (39.1)	311(39.2)	61 (32.1)	126 (41.2)	84 (41.8)	
<100	735 (49.3)	384 (48.4)	114 (60.0)	141 (46.1)	96 (47.8)	
**HIV viral load (log copies/mL), median (IQR)** ^**b**^	4.8 (4.4-5.3)	4.8 (4.4-5.2)	5.0 (4.6-5.4)	4.8 (4.3-5.2)	4.8 (4.3-5.2)	0.003
**Haemoglobin (g/dL)** ^**c**^						
Median, IQR	11.2 (9.8-12.5)	11.3 (10.0-12.6)	10.5 (9.3-12.3)	11.3 (10.0-12.3)	10.9 (9.7-12.2)	0.028
**WHO anaemia severity** ^**c**^						
None	318 (27.9)	185 (30.4)	33 (21.9)	66 (29.0)	34 (22.4)	0.048
Mild	304 (26.7)	163 (26.8)	34 (22.5)	67 (29.4)	40 (26.3)	
Moderate	469 (41.1)	235 (38.6)	74 (49.0)	85 (37.3)	75 (49.3)	
Severe	49 (4.3)	26 (4.3)	10 (6.6)	10 (4.4)	3 (2.0)	
**TB disease status**						
Past history of TB	710 (46.7)	368 (45.5)	116 (60.4)	133 (42.4)	93 (45.4)	0.001
Prevalent TB	452 (29.7)	211 (26.1)	72 (37.5)	103 (32.8)	66 (32.2)	0.005^#^
Any incident TB	423 (27.8)	232 (28.6)	59 (30.7)	83 (26.5)	49 (23.9)	0.405
Any TB ever (including past history, prevalent and incident TB)	1,147(75.4)	599 (74.0)	165 (85.9)	229 (72.9)	154 (75.1)	0.004

**Abbreviations:** *IQR* interquartile range, *TB* tuberculosis, *WHO* World Health Organization.

^a^31 missing values, ^b^101 missing values, ^c^ 381 missing values.

All values are numbers (%) unless otherwise stated.

^**¥**^ *P*-value tests for a difference across all groups; ^#^ *P*-value < 0.05 for a difference between those who were LTFU during ART and those who remained alive and in programme. There was no evidence to suggest a difference (*P* < 0.05) between any baseline characteristics of those who transferred out of the ART programme and those who remained alive and in programme.

### Incident TB diagnoses and TB incidence rates during ART

Overall, 476 episodes of incident TB were diagnosed in 423 (27.8%) patients during ART. Of these, 73.3% of episodes were culture-confirmed. After excluding 520.1 years of person-time accrued during TB treatment, the overall TB incidence rate during ART was 8.0 cases/100 PYs (95%CI, 7.3-8.8). TB incidence during the first 4 months of ART was more than fourfold higher (incidence rate ratio (IRR) = 4.1 [95%CI, 3.2-5.2]) than the rate during the remainder of ART follow-up (Table 2). Analysis restricted to culture-proven TB cases, yielded a slightly lower overall TB incidence rate (5.9 [95%, 5.3-6.6] cases/100 PYs).

**Table 2:** **TB incidence rates during ART stratified according to time-updated anaemia severity and CD4 cell count**

	**Overall TB incidence**			**TB incidence in first 4 months of ART**			**TB incidence after 4 months of ART**		
	**Total number**	**PY at risk**	**Rate per 100**	**Total number**	PY at risk*	**Rate per 100**	**Total number**	PY at risk*	**Rate per 100**
			**PYs (95%CI)**			**PYs (95%CI)**			**PYs (95%CI)**
**Updated WHO Anaemia severity**									
None	183	4,199.9	4.4 (3.8-5.1)	17	101.4	16.8 (10.6-28.2)	166	4,098.5	4.1 (3.5-4.7)
Mild	113	1,131.0	10.0 (8.3-12.1)	23	85.1	27.0 (18.1-42.1)	90	1,045.9	8.6 (7.0-10.7)
Moderate	154	578.6	26.6 (22.5-31.7)	39	111.2	35.1 (25.8-48.9)	115	467.4	24.6 (20.2-30.1)
Severe	26	29.6	87.8 (57.0-138.2)	8	11.2	71.7 (36.0-159.2)	18	18.5	97.5 (57.1-171.8)
**Updated CD4 cell count (cells/uL)**									
≥500	67	1,763.1	3.8 (3.0-4.9)	0	1.8	0	67	1,761.1	3.8 (3.0-4.9)
200-499	200	3,055.9	6.5 (5.7-7.6)	5	47.8	10.5 (4.4-31.0)	195	3,008.1	6.5 (5.6-7.5)
100-199	104	827.2	12.6 (10.4-15.4)	29	132.1	21.9 (15.4-32.4)	75	695.1	10.8 (8.6-13.7)
<100	105	292.9	35.8 (29.6-43.8)	53	127.0	41.7 (32.0-55.3)	52	165.9	31.3 (23.9-41.6)
**Total**	476	5,939.1	8.0 (7.3-8.8)	87	308.8	28.2 (22.9-35.0)	389	5,630.0	6.9 (6.2-7.7)

**Abbreviations:** *ART* antiretroviral therapy, *CI* confidence interval, *TB* tuberculosis; *WHO* World Health Organization.

*Person-time accrued during treatment of prevalent or incident TB (520.1 PYs) was excluded when calculating TB incidence rates.

### Relationship between TB incidence rates and time-updated anaemia severity

**Figure 2:**
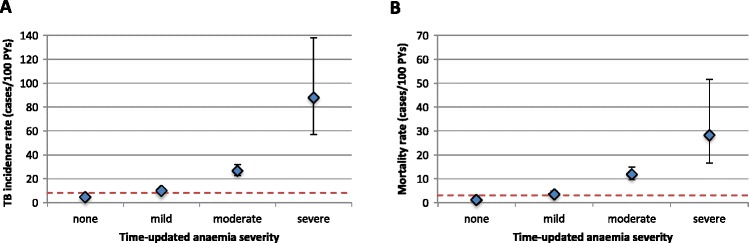
**Overall (A) TB incidence rates and (B) mortality rates during ART stratified according to time-updated WHO anaemia severity (cases/100 PYs).** The endpoints represent the corresponding 95% confidence intervals. The red, dashed line represents the overall rate among all patients (n = 1,521).

TB incidence rates were strongly and directly associated with time-updated anaemia severity (Figure 2A). Overall incidence rates ranged from 4.4 to 87.8 cases/100 PYs (20-fold difference) among those with no anaemia and severe anaemia, respectively (Table 2). TB incidence rates were 2.5-fold higher in patients with severe anaemia compared to incidence rates in patients with CD4 counts <100 cells/uL (Table 2). Although TB incidence rates were higher during the first 4 months of ART, rates were strongly and directly related to time-updated anaemia severity, regardless of ART duration (Table 2). The strong and direct association between TB incidence rates and time-updated anaemia severity was unchanged when analysis was restricted to culture-confirmed cases (data not shown).

### Independent predictors for incident TB during ART

We next sought to determine independent predictors for incident TB. In univariable analysis, several factors including time-updated anaemia severity were associated with incident TB during ART (Table 3). In multivariable analysis, time-updated moderate anaemia (adjusted IRR = 3.8 [95%CI, 3.0-4.8]) and severe anaemia (adjusted IRR = 8.2 [5.3-12.7]) were the strongest independent predictors of incident TB, although increasing age, time-updated CD4 cell counts, HIV viral load and a past medical history of TB were also independently associated with incident TB. When time-updated haemoglobin levels were coded as a continuous variable in the same multivariable model, they remained strongly predictive for incident TB (adjusted IRR = 1.29 [95%CI, 1.23-1.36] for each 1 g/dL decrease). Substituting baseline anaemia severity in place of time-updated anaemia severity in the above model revealed that baseline anaemia severity was not associated with incident TB during ART after correcting for potential confounders (data not shown).

**Table 3:** **Poisson regression of risk factors for incident TB among patients receiving ART**

	**Unadjusted IRR (95%CI)**	***P*** -value*	**Adjusted IRR (95%CI)**	***P*** -value*
**Age (for each year decrease)**	1.01 (1.00-1.02)	0.091	1.01 (1.00-1.03)	0.018
**Gender**				
Female	1.0	0.458		
Male	1.08 (0.89-1.31)			
WHO stage at baseline**		0.003		
1/2	1.0		1.0	0.457
3/4	1.44 (1.12-1.84)		1.10 (0.85-1.44)	
**Baseline anaemia severity**				
None	1.0	0.067		
Mild	0.96 (0.72-1.27)			
Moderate	1.27 (0.99-1.62)			
Severe	1.42 (0.87-2.32)			
**Baseline CD4 category (cells/μL)**				
≥150	1.0	0.298		
100-149	1.25 (0.96-1.63)			
50-99	1.21 (0.93-1.57)			
<50	1.22 (0.94-1.58)			
**Baseline viral load (cells/mL)**				
<5.0	1.0	0.984		
≥5.0	1.00 (0.96-1.04)			
CD4 category (cells/μL) (updated)**				
≥500	1.0	<0.001	1.0	<0.001
200-499	1.65 (1.25-2.18)		1.49 (1.11-1.99)	
100-199	2.93 (2.16-3.99)		2.02 (1.41-2.89)	
<100	6.63 (4.88-9.00)		3.15 (2.10-4.73)	
Viral load (cells/mL) (updated)**				
<1,000	1.0	<0.001	1.0	0.005
≥1,000	3.31 (2.74-4.00)		1.48 (1.13-1.94)	
WHO anaemia severity (updated)**				
None	1.0	<0.001	1.0	<0.001
Mild	2.15 (1.70-2.71)		1.98 (1.56-2.51)	
Moderate	5.011 (4.13-6.33)		3.80 (3.00-4.80)	
Severe	13.61 (9.02-20.52)		8.19 (5.29-12.69)	
**Duration of ART (months)**				
<4	1.0	<0.001	1.0	0.476
4-12	0.46 (0.34-0.63)		1.15 (0.81-1.63)	
12-24	0.35 (0.26-0.47)		1.35 (0.94-1.93)	
24-36	0.33 (0.24-0.45)		1.38 (0.95-2.01)	
36-48	0.34 (0.25-0.46)		1.47 (1.01-2.14)	
48-60	0.29 (0.20-0.40)		1.30 (0.86-1.95)	
>60	0.26 (0.18-0.37)		1.16 (0.75-1.79)	
**Past history of TB**				
No	1.0	<0.001	1.0	<0.001
Yes	1.47 (1.23-1.77)		1.55 (1.28-1.89)	
**Prevalent TB**				
No	1.0	0.473		
Yes	0.93 (0.75-1.14)			
**Recurrent TB**				
No	1.0	0.104		
Yes	1.18 (0.96-1.46)			

**Abbreviations:** *ART* antiretroviral, *IRR* incident rate ratio, *TB* tuberculosis, *WHO* World Health Organization.

*P-values calculated using likelihood ratio tests.

**No evidence for interaction between time-updated anaemia severity and time-updated CD4 count, time-updated viral load, duration of ART, WHO stage at ART initiation.

### Relationship between mortality and time-updated anaemia severity during ART

Overall, 192 (12.6%) patients died a median of 0.8 year after starting ART (IQR, 0.2-2.6). The overall mortality rate among patients receiving ART was 3.0 (95%CI, 2.6-3.5) cases/100 PYs. One-third (n = 64) of all deaths occurred in the first 4 months of ART, yielding a mortality rate of 14.0 (95%CI, 11.0-18.1) cases/100 PYs compared to 2.1 (95%CI, 1.8-2.6) cases/100 PYs thereafter (IRR = 6.5 [95%CI, 4.8-8.8]).

Mortality rates during ART were strongly associated with greater degrees of time-updated anaemia severity (Figure 2B), and those with severe anaemia had higher mortality rates throughout ART compared to those with advanced immunodeficiency (Table 4). Similar to TB incidence, mortality rates were higher in the first 4 months of ART; however, higher mortality rates were associated with greater anaemia severity regardless of ART duration (Table 4). In a sensitivity analysis, when all LTFU patients (n = 314) were presumed to have died (n = 506 total), the overall mortality rate was 7.8 (95%CI, 7.2-8.6) and the graded association with time-updated anaemia severity remained. Patients survived a median of 49 days (IQR, 26-88) from their last laboratory measurement to death, and this did not differ by time-updated anaemia severity or CD4 counts (data not shown).

**Table 4:** **Mortality rates stratified according to time-updated anaemia severity and CD4 cell count**

	**Overall Mortality**			**Mortality in first 4 months of ART**			**Mortality after 4 months of ART**			**Mortality <6 months after incident TB diagnosis**		
	**Total number**	**PY at risk**	**Rate per 100 PYs (95%CI)**	**Total number**	**PY at risk**	**Rate per 100 PYs (95%CI)**	**Total number**	**PY at risk**	**Rate per 100 PYs (95%CI)**	**Total number**	**PY at risk**	**Rate per 100 PYs (95%CI)**
**Anaemia severity**												
None	48	4,415.7	1.1 (0.8-1.5)	8	140.6	5.7 (2.9-12.8)	40	4,275.1	0.9 (0.7-1.3)	3	71.5	4.2 (1.3-20.6)
Mild	45	1,270.3	3.5 (2.7-4.8)	12	126.5	9.5 (5.5-17.8)	33	1,143.8	2.9 (2.1-4.2)	5	40.8	12.3 (5.1-36.8)
Moderate	86	727.0	11.8 (9.5-14.8)	39	172.0	22.7 (16.7-31.5)	47	555.0	8.5 (6.3-11.5)	22	45.4	48.5 (32.0-76.0)
Severe	13	46.1	28.2 (16.5-51.5)	5	18.1	27.6 (11.7-80.5)	8	28.0	28.5 (14.4-63.6)	4	6.9	58.4 (22.9-190.9)
**CD4 cell count (cells/uL)**												
≥500	13	1,809.0	0.7 (0.4-1.3)	0	2.7	0	13	1,806.3	0.7 (0.4-1.3)	4	22.4	0
200-499	48	3,264.8	1.5 (1.1-2.0)	4	69.4	5.8 (2.2-20.6)	44	3,195.4	1.4 (1.0-1.9)	11	74.7	9.4 (4.5-22.4)
100-199	44	957.5	4.6 (3.4-6.3)	14	177.7	7.9 (4.8-14.0)	30	779.7	3.8 (2.7-5.6)	3	36.2	22.1 (10.7-50.9)
<100	87	427.9	20.3 (16.5-23.4)	46	207.4	22.2 (16.7-30.0)	41	220.5	18.6 (13.6-25.8)	5	31.2	60.9 (39.6-97.2)
**Total**	192	6,459.2	3.0 (2.6-3.5)	64	457.2	14.0 (11.0-18.1)	128	6,001.9	2.1 (1.8-2.6)	34	164.5	20.7 (14.8-29.7)

### Mortality rates following episode of incident TB

Of 192 total deaths, 34 (17.7%) occurred within 6 months of an incident TB diagnosis, yielding a mortality rate of 20.7 cases/100 PYs (95%CI, 14.8-29.7). Mortality rates within 6 months of an incident TB diagnosis were strongly associated with time-updated anaemia severity (Table 4).

### Independent predictors for mortality during ART

Multivariable regression was undertaken to determine factors associated with mortality during ART (Table 5). Univariable analysis demonstrated several associations with mortality, including time-updated anaemia severity and incident TB. In multivariable analysis, several variables demonstrated independent associations with mortality during ART, including: increasing age, male gender, WHO disease stage at baseline, time-updated CD4 cell count, time-updated anaemia severity, time-updated viral load, prevalent TB at ART initiation and time-updated incident TB. However, the strongest independent predictor of mortality was time-updated severe anaemia (adjusted IRR = 8.0 [95%CI, 3.9-16.4]), while time-updated moderate anaemia (adjusted IRR = 6.0 [95%CI, 3.9-9.2]) and CD4 counts <100 cells/uL (adjusted IRR = 6.5 [95%CI, 2.8-15.0]) were also very strong independent predictors. Time-updated haemoglobin levels retained a strong relationship with mortality risk when coded as a continuous variable (adjusted IRR = 1.49 [95%CI, 1.36-1.63] for each 1 g/dL decrease). When a combined outcome of either mortality or LTFU (n = 506) was used, time-updated severe anaemia remained the strongest independent predictor (adjusted IRR = 4.2 [95%CI, 2.6-6.7]) and time-updated moderate anaemia (adjusted IRR = 2.5 [95%CI, 1.9-3.2]) and time-updated CD4 counts <100 cells/uL (adjusted IRR = 2.5 [95%CI, 1.7-3.8]) were also strong independent predictors, while time-updated HIV viral load, male gender, prevalent TB and incident TB also demonstrated smaller independent associations (data not shown).

**Table 5:** **Poisson regression of risk factors for mortality among patients receiving ART**

	**Unadjusted IRR (95%CI)**	***P*** -value*	**Adjusted IRR (95%CI)**	***P*** -value*
**Age (for each year increase)**	1.04 (1.02-1.06)	<0.001	1.04 (1.02-1.06)	<0.001
**Gender**				
Female	1.0	<0.001	1.0	0.002
Male	1.69 (1.25-2.29)		1.79 (1.21-2.66)	
**WHO stage at baseline**				
1/2	1.00	<0.001	1.0	0.011
3/4	3.34 (1.93-5.78)		2.06 (1.12-3.78)	
**Baseline anaemia severity**				
None	1.0	0.025		
Mild	1.10 (0.67-1.80)			
Moderate	1.66 (1.09-2.53)			
Severe	2.28 (1.08-4.85)			
**Baseline CD4 category (cells/μL)**				
≥150	1.0	0.011		
100-149	1.05 (0.66-1.67)			
50-99	1.20 (0.77-1.86)			
<50	1.83 (1.22-2.75)			
**Baseline viral load (cells/mL)**				
<5.0	1.0	0.007		
≥5.0	1.51 (1.12-2.05)			
CD4 category (cells/μL) (updated)**				
≥500	1.0	<0.001	1.0	<0.001
200-499	2.05 (1.11-3.78)		1.38 (0.71-2.68)	
100-199	6.39 (3.44-11.88)		2.75 (1.28-5.89)	
<100	27.98 (15.59-50.22)		6.50 (2.82-15.00)	
Viral load (cells/mL) (updated)**				
<1,000	1.0	<0.001	1.0	0.034
≥1,000	6.91 (5.18-9.22)		1.62 (1.04-2.53)	
WHO anaemia severity (updated)**				
None	1.0	<0.001	1.0	<0.001
Mild	3.19 (2.11-4.81)		2.42 (1.55-3.77)	
Moderate	10.89 (7.57-15.64)		6.03 (3.93-9.24)	
Severe	25.94 (13.90-48.40)		7.99 (3.89-16.44)	
Duration of ART (months)**				
<4	1.0	<0.001	1.0	0.843
4-12	0.35 (0.24-0.52)		1.30 (0.77-2.16)	
12-24	0.15 (0.10-0.24)		1.07 (0.55-2.07)	
24-36	0.13 (0.08-0.21)		0.96 (0.46-1.99)	
36-48	0.13 (0.08-0.22)		1.07 (0.46-2.46)	
48-60	0.09 (0.05-0.17)		0.83 (0.32-2.17)	
>60	0.08 (0.04-0.17)		0.78 (0.27-2.23)	
**Current AZT use**				
No	1.0	<0.001	1.0	0.501
Yes	0.38 (0.27-0.54)		0.86 (0.56-1.33)	
**Prevalent TB**				
No	1.0	<0.001	1.0	0.024
Yes	1.72 (1.27-2.33)		1.50 (1.06-2.14)	
**Incident TB**				
No	1.0	<0.001	1.0	<0.001
Yes	1.85 (1.34-2.55)		2.23 (1.47-3.37)	

**Abbreviations:** *ART* antiretroviral, *TB* tuberculosis, *WHO* World Health Organization, *IRR* incident rate ratio.

*P-values calculated using likelihood ratio tests.

**No evidence for interaction between time-updated anaemia severity and time-updated CD4 count, time-updated viral load, duration of ART, WHO stage at ART initiation.

## Discussion

In this prospective cohort of HIV-infected patients receiving ART for up to 8 years in South Africa, haemoglobin levels had high predictive value for incident TB and death. Both TB incidence rates and mortality rates were strongly and directly associated with time-updated anaemia severity, regardless of ART duration. We found that time-updated moderate or severe anaemia during ART were the strongest independent predictors for both incident TB and death during ART and, notably, were of greater predictive value than CD4 cell counts.

Patients with a recent haemoglobin measurement (within at least the past 4 months) indicating either moderate or severe anaemia during ART were at very high risk for incident TB, in agreement with previous studies from South Africa and Cambodia [3,20]. Additionally, it has been found that anaemia prior to ART initiation is highly predictive for undiagnosed prevalent TB or early incident TB during ART [3,11,21]. However, because haemoglobin levels are dynamic and can change quickly, this may explain why this and other studies have found that baseline anaemia prior to or at ART initiation has poor predictive value for incident TB during ongoing ART [22,23]. Therefore, only current WHO anaemia severity appears to have predictive value for incident TB disease during ART.

TB incidence rates during ART were so high and the independent associations so strong, that routine microbiological investigation for TB among those found to have moderate/severe anaemia (haemoglobin concentration <11.0 g/dL) during ART in high incidence areas should be considered. Additionally, mortality rates within 6 months of an incident TB diagnosis were exceedingly high among those with recent moderate/severe anaemia. Thus, routine screening for TB among those with moderate/anaemia may help reduce mortality by expediting initiation of appropriate treatment. Recent studies have demonstrated that the sensitivities of Xpert MTB/RIF assay on sputum and urine, sputum AFB microscopy and urine lipoarabinomannan (LAM) lateral-flow assay for the diagnosis of HIV-associated TB are significantly greater among those with moderate/severe anaemia compared to those with no/mild anaemia [11,24,25]. When TB is confirmed among those with HIV-related anaemia, it is reassuring that most can expect haemoglobin recovery with combined ART and anti-TB treatment without specific additional interventions for anaemia [26].

While strong, independent relationships between anaemia and incident TB were found, the biology underpinning these relationships is complex. Haemoglobin levels were measured at regular intervals throughout ART (as opposed to TB investigations, which were undertaken when clinically indicated), and thus more severe anaemia was likely in large part due to yet undiagnosed active TB, resulting in pro-inflammatory cytokine up-regulation and subsequent anaemia of chronic disease. However, emerging evidence suggests that the dysregulated iron axis in HIV-related anaemia may provide highly favourable intracellular macrophage conditions for TB bacilli and the subsequent development of TB disease [27,28]. Thus, while anaemia during ART most likely represents a downstream consequence of active TB, we cannot exclude the possibility that it may also represent an upstream risk factor for active TB.

Mortality rates during ART were strongly associated with time-updated anaemia severity, and moderate or severe anaemia were the strongest independent predictors of death during ART. This is consistent with other studies from Africa that show that anaemia either before or during ART has high prognostic value [29-38] and suggests a need to develop standardized guidelines for the management of HIV-related anaemia to help reduce associated mortality. ART should be considered a primary intervention for HIV-related anemia, as it may resolve in anaemia in a majority of patients [26,39-43]. Additionally, management of those with anaemia during ART might include a greater frequency of follow-up in care, especially during the first 3-6 months of ART (given very high mortality rates), to allow for assessment of the patient’s current health and need for adjunctive interventions. Anaemia may serve as a crude marker for virological failure [44-46], and thus requesting an HIV viral load measurement may be warranted to determine if a patient is responding appropriately to ART. In addition to routine screening for TB and initiation of anti-TB therapy as described above, additional interventions a clinician may consider for those with anaemia during ART might include: treatment of comorbid infections that may contribute to anaemia (parasites, opportunistic infections), switching or stopping drugs that may be associated with anaemia (zidovudine, trimethoprim-sulphamethoxazole, among others), correction of nutritional deficiencies (iron, folate, vitamin B12), and investigations for AIDS-related malignancies. Of note, most patients survived for more than 45 days from the date of their final haemoglobin measurement to death, suggesting that there is a sufficient window of time to investigate the causes underlying HIV-related anaemia during ART and to implement appropriate interventions that may impact mortality risk.

Given the high predictive value of anaemia for both incident TB and mortality, this study demonstrates the potential utility of regular haemoglobin measurements both prior to ART initiation and at regular intervals during treatment. While automated haematologic analysers are the gold standard for determining haemoglobin concentrations, they are not routinely available for use in resource-limited settings. The WHO Haemoglobin Colour Scale is a simple, paper-based assay costing less than 15 cents per test [47], but the test has yielded mixed results in several studies, with overall moderate sensitivity and generally poor specificity [48]. The HemoCue system has been validated in resource-limited settings, is durable, easy to use and has shown good accuracy overall [49-51], but its cost and requirement for ongoing proprietary supplies may make its use inappropriate in some settings. In the near future, tests utilizing transcutaneous spectrophotometry may offer an inexpensive solution that allows for the routine measurement of haemoglobin levels in HIV-infected patients without a requirement for blood samples [52-54]. As methods for measuring haemoglobin become cheaper, more widely available and possibly less invasive, more frequent measurements during ART may be warranted and feasible.

This is the first study to report TB incidence and mortality rates during ART stratified according to time-updated WHO anaemia severity. Additional strengths include a large, consecutively recruited cohort followed up for a median of more than 5 years under routine programme conditions. While this study was conducted at a single site, it was a very large community-based cohort with comparable characteristics to other public sector ART cohorts in sub-Saharan Africa [7-9,55] and spanned a period of 8 years. Additionally, risk of selection bias was low due to limited inclusion/exclusion criteria and only about 1% of patients were excluded for not having haemoglobin results available. Our results are therefore likely generalizable to other ambulatory ART programmes in sub-Saharan Africa where there is a high TB burden.

There was a female predominance in our cohort; however, this likely reflects the epidemiology of HIV in sub-Saharan Africa, where the epidemic is well recognized as being feminized [56]. The proportion of female patients is entirely consistent with ART programmes throughout the region and would not be expected to affect the generalizability of our findings. Unfortunately, data were not available on pregnancy status, which may have led to a small minority of women having the severity of their anaemia misclassified during ART.

Also, we did not analyse the time lag between time-updated haemoglobin measurements and the diagnosis of incident TB; however, the vast majority of haemoglobin measurements were done at least 4-monthly. There was also a proportion of patients who were LTFU and for whom outcomes could not be ascertained, although this proportion is similar to other long-term ART cohorts in South Africa [3,57]. LTFU patient characteristics were very similar to those who remained alive throughout follow-up, and while LTFU patients may have a high mortality risk [57,58], this did not appear to apply to the present study. Furthermore, when such patients were presumed to have died, time-updated moderate or severe anaemia remained the strongest independent predictors of a poor clinical outcome.

## Conclusion

Time-updated anaemia severity had very high predictive value for both incident TB and mortality during ART. Haemoglobin levels should be determined prior to ART initiation and at routine intervals during ART. Moderate or severe anaemia during ART should prompt systematic investigation of TB using microbiological assays as well as adjunctive clinical interventions given the very high risk of death among such patients.
